# Enhanced human enterovirus 71 infection by endocytosis inhibitors reveals multiple entry pathways by enterovirus causing hand-foot-and-mouth diseases

**DOI:** 10.1186/s12985-017-0913-3

**Published:** 2018-01-03

**Authors:** Meichun Yuan, Jingjing Yan, Jingna Xun, Chong Chen, Yuling Zhang, Min Wang, Wenqi Chu, Zhigang Song, Yunwen Hu, Shuye Zhang, Xiaoyan Zhang

**Affiliations:** 1 Shanghai Public Health Clinical Center and Institutes of Biomedical Sciences, Fudan University, Shanghai, China; 2 Key Laboratory of Medical Molecular Virology of Ministries of Education/Health, Institute of Medical Microbiology, Shanghai Medical College of Fudan University, Shanghai, China; 3 Department of Scientific Research, Shanghai Emerging and Re-emerging infectious Diseases Institute, 2901 Caolang Road, Jin-Shan District, Shanghai, 201508 People’s Republic of China

**Keywords:** Clathrin, Dynamin, Endocytosis, Enterovirus, CV-A16

## Abstract

**Background:**

Human enterovirus 71 (EV71) was previously known to enter cells through clathrin or caveolar mediated endocytic pathways. However, we observed chlorpromazine (CPZ) or dynasore (DNS), which inhibit clathrin and dynamin mediated endocytosis, did not suppress EV71 cell entry in particular cell types. So the current knowledge of entry mechanisms by EV71 is not complete.

**Methods:**

Viral infection was examined by flow cytometry or end-point dilution assays. Viral entry was monitored by immunofluorescence or pseudoviral infections. Various inhibitors were utilized for manipulating endocytic pathways. Cellular proteins were knockdown by siRNA.

**Results:**

CPZ and DNS did not inhibit but rather enhance viral infection in A549 cells, while they inhibited infections in other cells tested. We further found CPZ did not affect EV71 binding to target cells and failed to affect viral translation and replication, but enhanced viral entry in A549 cells. Immunofluorescence microscopy further confirmed this increased entry. Using siRNA experiment, we found that the enhancement of EV71 infection by CPZ did not require the components of clathrin mediated endocytosis. Finally, CPZ also enhanced infection by Coxackivirus A16 in A549 cells.

**Conclusions:**

CPZ and DNS, previously reported as EV71 entry inhibitors, may rather lead to increased viral infection in particular cell types. CPZ and DNS increased viral entry and not other steps of viral life cycles. Therefore, our study indicated an unknown dynamin-independent entry pathway utilized by enteroviruses that cause Hand-Foot-and-Mouth Diseases.

**Electronic supplementary material:**

The online version of this article (10.1186/s12985-017-0913-3) contains supplementary material, which is available to authorized users.

## Background

Hand-foot-and-mouth disease (HFMD) is a common, usually self-limiting illness affecting large numbers of infants and young children worldwide [1]. The clinical symptoms include fever, vesicular rashes on hands, feet, buttocks and the mouth. However, a small proportion of infected children may suffer severe complications including meningitis, acute flaccid paralysis, neurorespiratory syndrome and even fatal illness. The most important causative agents for HFMD are the enterovirus 71 (EV71) and coxackievirus A16 (CV-A16). The severe outcomes are often seen in EV71 infections but unusual with CV-A16. Recently, HFMD associated with other enteroviruses also emerged, including the coxackievirus A6 (CV-A6) and coxackievirus A10 (CV-A10) [1].

EV71 is a member of the species *Enterovirus A*, genus *Enterovirus* within the family *Picornaviridae*. The structure of the EV71 virion has been resolved recently, which showed an icosahedral shell with typical features of enteroviruses [2, 3]. The EV71 mature virion has a canyon around the five-fold axes, probably as the binding site for EV71 specific receptor. Several receptors have been reported, however, only human scavenger receptor class B, member 2 (SCARB2) was subsequently shown to induce uncoating of EV71 in vitro [4]. Recent studies suggested that the head region of SCARB2 comprising a pH-sensing three α-helix bundle might bind to the canyon and expel of the pocket factor from the EV71 virion, hence destabilizing the capsid and triggering the uncoating process [5]. In addition to SCARB2, P-selectin glycoprotein ligand-1 (PSGL1), Annexin2, heparan sulfate, sialylated glycans, and nucleolin were also identified as EV71 binding receptors [6–10]. However, these molecules cannot induce uncoating, so their roles may be related to capture EV71 on the cell surface and deliver it to SCARB2 in the endosomes.

Investigation of endocytic mechanisms revealed that clathrin mediated endocytosis (CME) was responsible for EV71 entry into human rhabdomyosarcoma cells (RD cells). Knockdown of the components in CME by small interfering RNAs (siRNAs) significantly inhibited EV71 infection. The dominant negative mutants of CME pathway, inhibitors of CME pathway and neutralizers of endosome acidification also reduced EV71 entry into RD cells [11]. In addition, overexpression of human SCARB2 facilitated EV71 infection in a murine cell line, NIH/3 T3 cells. However, blocking CME by siRNA inhibited EV71 infection in the SCARB2-expressing NIH/3 T3 cells [12]. Overall, these data suggest that EV71 may enter various cells via the CME dependent pathway and uncoat in an acidified endocytic compartment. Another study showed that EV71 could enter Jurkat cells (human T leukemia cell line) and PSGL-1-expressing L929 cells via the caveolar-dependent endocytic pathway (CDE) when binding to PSGL-1 [13]. Both CME and CDE entry pathways require the dynamin to segregate the endocytic vesicles from the cell surface membrane. Thus, endocytosis inhibitors, including chlorpromazine [14] and dynamin [15] have been proposed or attempted to prevent or treat microbial infections.

Despite of the importance of SCARB2 in EV71 infection, several evidences indicate that the SCARB2-independent EV71 entry may exist as well. Normally, SCARB2 mainly localizes in the endosomal/lysosomal membrane, responsible for delivery of β-glucocerebrosidase from the endoplasmic reticulum to lysosomes, where the enzyme degrades glucocerebroside and its gene mutation causes defective metabolism of glucocerebrosides and Gaucher disease [16, 17]. Only small amount of SCARB2 may shuttle to the plasma membrane as EV71 receptor. Thus, the EV71 binding and early entry very likely depend on other non-SCARB2 receptors. Second, SCARB2 transgenic mice study showed that not all tissues supported viral replication despite of their SCARB2 expression, suggesting some SCARB2-independent mechanisms contribute to EV71 susceptibility or permissiveness [18]. Thirdly, one report found that knockdown of SCARB2 prevented EV71 infection in RD cells but not in Vero cells and concluded that other unidentified molecules functioned as EV71 receptors [12]. Finally, although CV-A16 utilizes SCARB2 as its receptors, other major HFMD associated enteroviruses, CV-A6 and CV-A10 did not [19]. Therefore, EV71 may enter host cells using other non-SCARB2 receptors and their endocytic pathways are unclear yet.

Here, to further examine the features of EV71 infection, several cell lines were examined in a pilot study. Although EV71 could establish infection in most of them, the viral kinetics and spreading patterns in different cells were clearly distinct. Importantly, we found that the known EV71 entry inhibitors, including both cholopromazin and dynasore, unexpectedly led to enhanced EV71 infection in A549 cells, indicating the existence of a clathrin and dynamin-independent endocytic pathway for EV71 in human lung epithelial A549 cells.

## Methods

### Cells and Viruses.

Human lung epithelial A549 cells were cultured in RPMI 1640 supplemented with 10% fetal bovine serum (FBS, Gibco, Waltham, MA, USA), 100 U/ml penicillin, and 100 μg/ml streptomycin. Human rhabdomyosarcoma (RD) cells, human embryonic kidney 293 T (HEK293T) cells, human lung fibroblast MRC-5, African green monkey kidney Vero cells, human cervical epithelial Hela cells, human hepatoma epithelial cells Huh-7 and HepG2 cells were cultured in Dulbecco’s modified Eagle’s medium (DMEM) supplemented with 10% FBS, 100 U/ml penicillin, and 100 μg/ml streptomycin. All the cell lines were purchased from the Cell Bank of the Chinese Academy of Sciences (Shanghai, China). EV71 strain (FJ08089) [20] belonging to genotype C4 was originally isolated by Centers for Disease Control and Prevention in Fujian province and propagated in RD cells. The culture supernatants containing the virus were harvested when all cells were dying. After centrifugation, the virus was filtered, aliquoted and stored at −80 °C. Viral titers were determined in RD cells by end-point dilution assay.

### Antibodies and Reagents.

Mouse monoclonal antibody against EV71 capsid protein VP-1 was purchased from Abcam (Cambridge, UK). Rabbit monoclonal antibody to AP2M1 (Abcam), Rabbit polyclonal antibodies to FCHO2 (ProSci, Poway, CA, USA), eIF4G (Cell Signaling Technology, Danvers, MA, USA) and Enterovirus 71 2C and 3C (GeneTex, Irvine, CA, USA), Mouse monoclonal antibodies against clathrin (CLTC, Santa Cruz Biotechnology, Dallas, Texas, USA) and dynamin (Santa Cruz Biotechnology) were used in Western-blot detection of the respective proteins. The secondary antibody conjugated to Allophycocyanin (APC) was from BD Biosciences (Franklin Lakes, CA, USA). AlexaFluor594-Conjugated AffiniPure Goat Anti-Mouse IgG (H + L) was from ZSGB-BIO (Beijing, China). Fluoroshield Mounting Medium with DAPI was from Abcam. AlexaFluor 488 conjugated transferrin from human serum and AlexaFluor 594 conjugated cholera toxin subunit B were from ThermoFisher scientific (Waltham, MA, USA). Chloropromazin and dynasore were purchased from Selleckchem (Shanghai, China). Guanidine hydrochloride (GnHCl) was from MedChem Express (Monmouth Junction, NJ, USA). All drugs were prepared according to the manufacturer’s instructions under sterile conditions.

### Cell viability assay.

Cell viability upon siRNA transfection and drug treatment was assessed by employing the cell counting kit-8 (CCK-8, Dojindo Laboratories, Kumamoto, Japan). Briefly, cells were seeded in 96-well cell culture plates and subsequently treated with drugs for 12 h before incubation with 10 μl CCK-8 for 20 min at 37 °C. After this, the data of absorbance at a wavelength of 450 nm was collected to analyze cell viability.

### Small RNA interference and gene knockdown.

siRNA for clathrin heavy chain (CLTC, Gene ID: 1213), dynamin 2 (DNM2, Gene ID: 1785), adaptor related protein complex 2 mu 1 subunit (AP2M1, Gene ID: 1173), FCH domain only 2 (FCHO2, Gene ID: 115,548) and scrambled siRNA, which were used in RNA interference experiments, were purchased from Gene Pharma (Shanghai, China), and the sequences of siRNA are listed in Additional file: 1 Table S1. All transfections were performed in a 12-well plate format. 5 × 10^4^ RD or 1 × 10^5^ A549 cells per well were plated overnight. Then 2 μl transfection reagent and 2.5 μl siRNA (20 μM) were prepared in Opti-MEM Reduced Serum Media (200 μl/well, Gibco) and incubated at RT for 10 min to form siRNA-lipid complexes. Subsequently, 800 μl Opti-MEM and 200 μl siRNA-lipid complexes were added to the cells. The final concentration of pooled siRNAs was 50 nM. The gene knockdown efficiency was determined by real-time qRT-PCR at 48 h and by Western blotting at 96 h. The experiments of EV71 infection were conducted at day 4 after siRNA transfection.

### Monitoring viral infectivity by Flow cytometry.

EV71 or CV-A16 infected cells were trypsinized with 0.25% trypsin-EDTA and then collected for intracellular staining. Harvested cells were washed with 1% FBS-PBS, fixed and permeabilized with Fixation/Permeabilization solution (BD Biosciences) for 20 min, then washed with 1 × BD Perm/Wash buffer, incubated with the mouse anti-EV71 VP-1 antibody (diluted by 1: 1000, Abcam) at RT for 40 min, then washed and stained in 0.1 ml of APC-conjugated rat anti-mouse IgG1 (diluted by 1: 200, BD Biosciences) for 30 min, washed and resuspended in Perm/Wash buffer, finally analyzed by BD Accuri C6 (BD Biosciences).

### EV71 single round infection system.

The plasmids of EV71 subgenomic replicon and EV71 capsid were kindly provided by Prof. Wenhui Li from National Institute of Biological Sciences, Beijing (NIBS). EV71 replicon construct contains all the gene segments of EV71 except the capsid portion which is replaced by the firefly luciferase. This EV71 replicon construct was led by a T7 promoter in the plasmid, and the replicon RNA was obtained from the linearized plasmid by in vitro transcription. The vector information and detailed experimental procedures can be found in the published method [21]. In brief, the EV71 pseudotype viruses were produced by sequential transfection of capsid plasmid and EV71 replicon RNA into HEK293T cells. Capsid plasmid was firstly transfected into HEK293T cells at 60–80% confluence; 24 h later, replicon RNA was then transfected using lipofectamine 3000 (Invitrogen, Carlsbad, CA, USA). EV71 pseudotype was harvested 24 h post-RNA transfection with two rounds of freeze-thaw cycle.

50 μl of EV71 pseudotype was mixed with 450 μl of fresh DMEM and applied to cells in 24-well plate. 12 h later, 20 μM CPZ or DMSO was added. In addition, 0.5 μg/well EV71 replicon RNA was transfected into cells in 24-well plate. 12 h post-transfection, 20 μM CPZ or DMSO was applied. Firefly luciferase activity was measured following the Luciferase Assay System manual (Promega, Madison, WI, USA) at 24 h post-infection or post-transfection.

### Virus binding assay.

Cells were seeded in 12-well plates (3 × 10^5^ cells/well). The next day, cells were incubated with EV71 at an MOI of 50 and CPZ (20 μM) or DMSO for 2 h at 4 °C. Then cells were washed with cold PBS to remove unbound viruses. Total RNA was then extracted using the RNA purification kit (TIANGEN, Beijing, China). Reverse transcriptions were then carried out using the HiScript II first strand cDNA synthesis kit (Vazyme, Nanjing, China). SYRB Green-based qPCR was performed on the Eppendorf Realplex Mastercycler (Eppendorf, Hamburg, Germany) using GoTaq qPCR Master Mix (Promega). The primers for EV71 and h18 s quantification were as follows: EV71-F: 5′- GCAGCCCAAAACAACTTCAC-3′, EV71-R: 5′- AATTTCAGCAGCTTGGAGTGC-3′; h18 s-F: 5’-GTAACCCGTTGAACCCCATT-3′, h18 s-R: 5’-CCATCCAATCGGTAGTAGCG-3′.

### Viral entry assay by immunofluorescence microscopy.

A549 cells and RD cells were seeded in 12-well plates (3 × 10^5^ cells/well) with coverslips. The next day, A549 cells and RD cells were pretreated with 2.5 mM GnHCl for 30 min at 37 °C, and then incubated with EV71 at an MOI of 50 (A549 cells) or 10 (RD cells) at 4 °C for 2 h respectively. Then, A549 and RD cells were shifted to 37 °C and treated by 20 μM CPZ or DMSO for another 6 h. Thereafter, cells were washed with cold PBS, fixed with 4% paraformaldehyde (PFA, Sigma, St Louis, MO, USA) for 15 min, permeabilized with 0.05% Triton X-100 in 2% FBS/PBS, and then stained with mouse anti-EV71 VP-1 antibody. Three washes with PBS were followed by a 30 min-incubation in the dark with the secondary antibodies conjugated to AlexaFluor 594. After three washes with PBS, coverslips were applied to mounting medium with DAPI. Digital confocal imaging was performed on EVOS® FL Color Imaging Systems (Life technology, Grand Island, NY, USA).

### Western blotting.

Cells were first harvested and then lysed in Radioimmunoprecipitation assay buffer (RIPA buffer) with a mixture of protease inhibitors. The concentration of proteins was quantified by bicinchoninic acid (BCA) protein assay and equal amounts of proteins were loaded and separated by SDS-PAGE, then transferred to a nitrocellulose membrane. The membrane was blocked with 0.1% Tween-20 in PBS containing 5% BSA and then was incubated overnight with primary antibodies at 4 °C. The membrane was then washed three times in 0.1% Tween-20/PBS and incubated with anti-rabbit IgG conjugated to AlexaFluor680 (Jackson ImmunoResearch, West Grove, PA, USA) for 2 h at room temperature. The immunoblots were visualized using an Odyssey Fc Imager (Lincoln, NE, USA).

### Transferrin and Cholera Toxin B Uptake assay.

Transferrin is a common marker for clathrin-mediated endocytosis (CME) while Cholera Toxin B is the marker for caveolar-dependent endocytosis (CDE). To verify the inhibitory effects of CPZ and DNS, the inhibitors of CME and dynamin respectively, the uptake assay of fluorescence-labeled transferrin (through CME) and Cholera Toxin B (through CDE) were performed. Cells were seeded in 12-well plates with coverslips and then pretreated with 20 μM CPZ, 80 μM DNS or DMSO for 2 h. After treatment, cells were incubated on ice for 10 min and washed. Then, cells were incubated with 25 μg/ml AlexaFluor488 labeled human Transferrin or 10 μg/ml AlexaFluor594 labeled Cholera Toxin B for 15 min at 37 °C. Thereafter, cells were washed with PBS, fixed with 4% PFA for 15 min, and permeabilized with 0.05% Triton X-100 in 2%FBS/PBS. After wash, coverslips were applied to mounting medium with DAPI. Digital confocal imaging was performed on EVOS® FL Color Imaging Systems.

### Statistical analysis

Significance of differences was evaluated using paired Student’s *t* test. Statistical analysis was performed with GraphPad Prism version 6.0 (La Jolla, CA, USA).

## Results

### CPZ did not inhibit, but rather enhance EV71 infection in A549 cells.

**Fig. 1:**
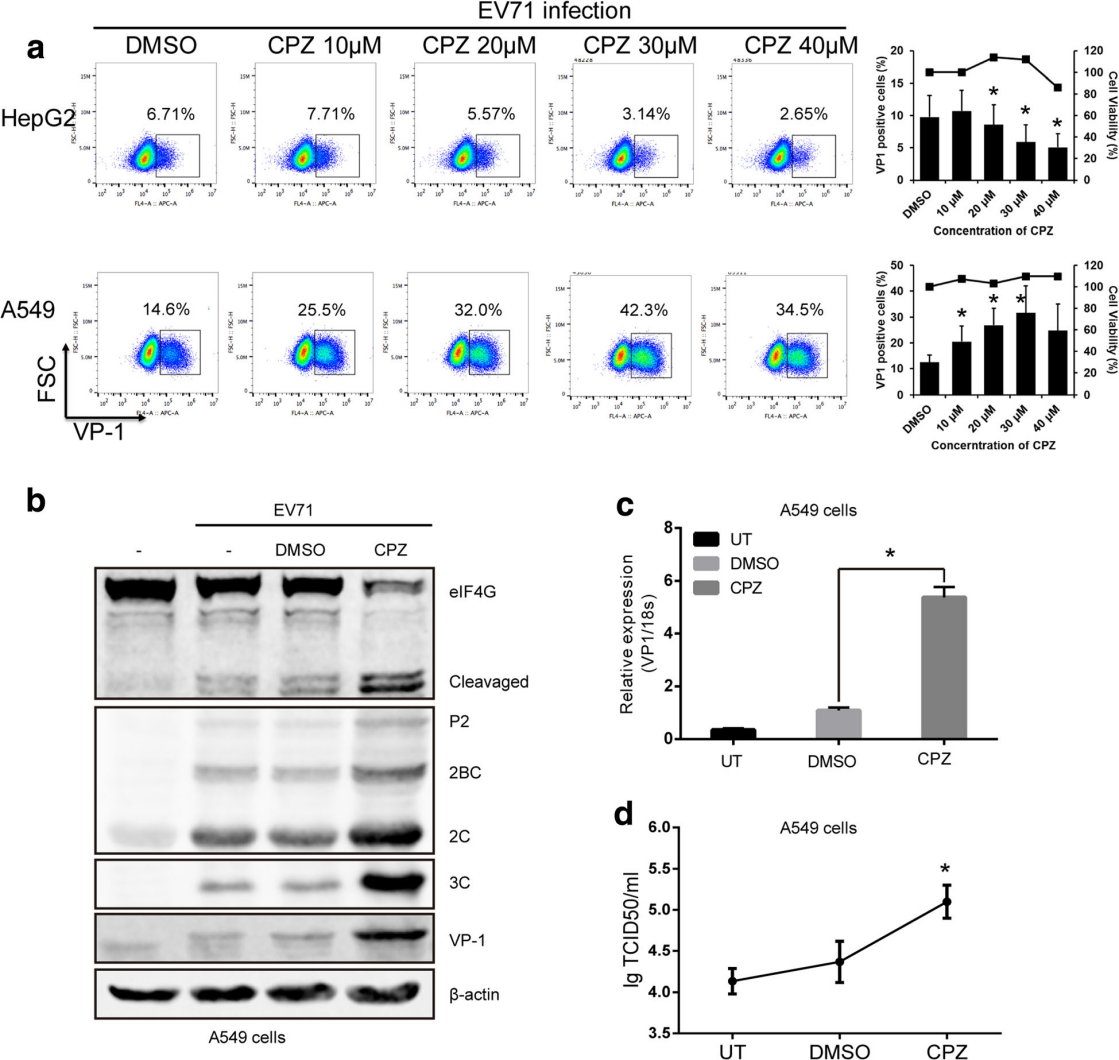
The effect of CPZ on EV71 infection in HepG2 and A549 cells. **a**. HepG2 and A549 cells were pretreated with increasing concentrations of CPZ (10, 20, 30 and 40 μM) for 2 h at 37 °C before EV71 infection. At 24 hpi, the infected cells were processed for flow cytometry. The bar charts represented the EV71 infectivity determined by the percentage of VP-1 positive cells and were shown as means with SD from three independent experiments. *, *p* < 0.05. Cell viability upon CPZ treatments was shown by the curve above the chart. **b-d**. A549 cells were infected with EV71 at an MOI of 5 for 12 h and then CPZ or DMSO was added. At 24 hpi, cells were lysed, and eIF4G cleavage, EV71- 2C, 3C and VP-1 were determined by Western blot (**b**); EV71 VP-1 mRNA levels were measured by qRT-PCR and normalized to 18 s (**c**); Virus titers were determined and shown in TCID50 (**d**). The blot shown was representative of three independent experiments and the full-length bolts were presented in Additional file 1 Fig. S5. Results were presented as the mean with SD from three independent experiments. *, *p* < 0.05. UT, untreated cells; TCID50, 50% tissue culture infective dose

Chlorpromazine (CPZ) is a known inhibitor of clathrin-mediated endocytosis. Pretreatment with increasing concentrations of CPZ revealed significant dose-dependent inhibition of EV71’s infectivity in HepG2 cells, shown as the decreased levels of viral capsid protein VP-1. Almost 50% inhibition by 30 μM CPZ was observed in this experiment. Surprisingly, we found that the infection of EV71 in A549 cells was rather enhanced when treated with increasing concentrations of CPZ (Fig. 1a). In contrast, pretreatment of EV71 by CPZ showed on effect on subsequent infections in A549 cells (Fig. S1). Possible drug-induced cytotoxic effects were assessed by cell viability assays and showed no obvious cytotoxicity.

The initiation factor of cap-dependent translation eIF4G will be cleaved during the course of EV71 infection. After invasion of targeted cells, the 2A proteinase of EV71 is expressed and causes cleavage of eIF4G, which will stop the cellular cap-dependent translation [22]. Therefore, eIF4G cleavage represents the early events during EV71 infection. To further determine this unusual effect of CPZ on A549 cells, we measured the levels of eIF4G cleavage, as well as the expression of non-structural (2C and 3C) and structural viral protein (VP-1) by Western blot after CPZ treatment. Our results showed both eIF4G cleavage and expression of EV71 proteins were largely increased in A549 cells when treated with CPZ, comparing to DMSO (Fig. 1b). In addition, measurement of RNA levels also confirmed the enhanced effect of CPZ (Fig. 1c). Finally, the virus titer was higher after CPZ treatment (Fig. 1d). Since VP-1 expression and virus titer were correlated, VP-1 staining was adopted to monitor viral infection in the following study. These data confirmed that CPZ indeed enhanced EV71 infection in A549 cells, although inhibited infection in HepG2 cells.

### The enhanced effect of CPZ is independent of dynamin activity.

**Fig. 2:**
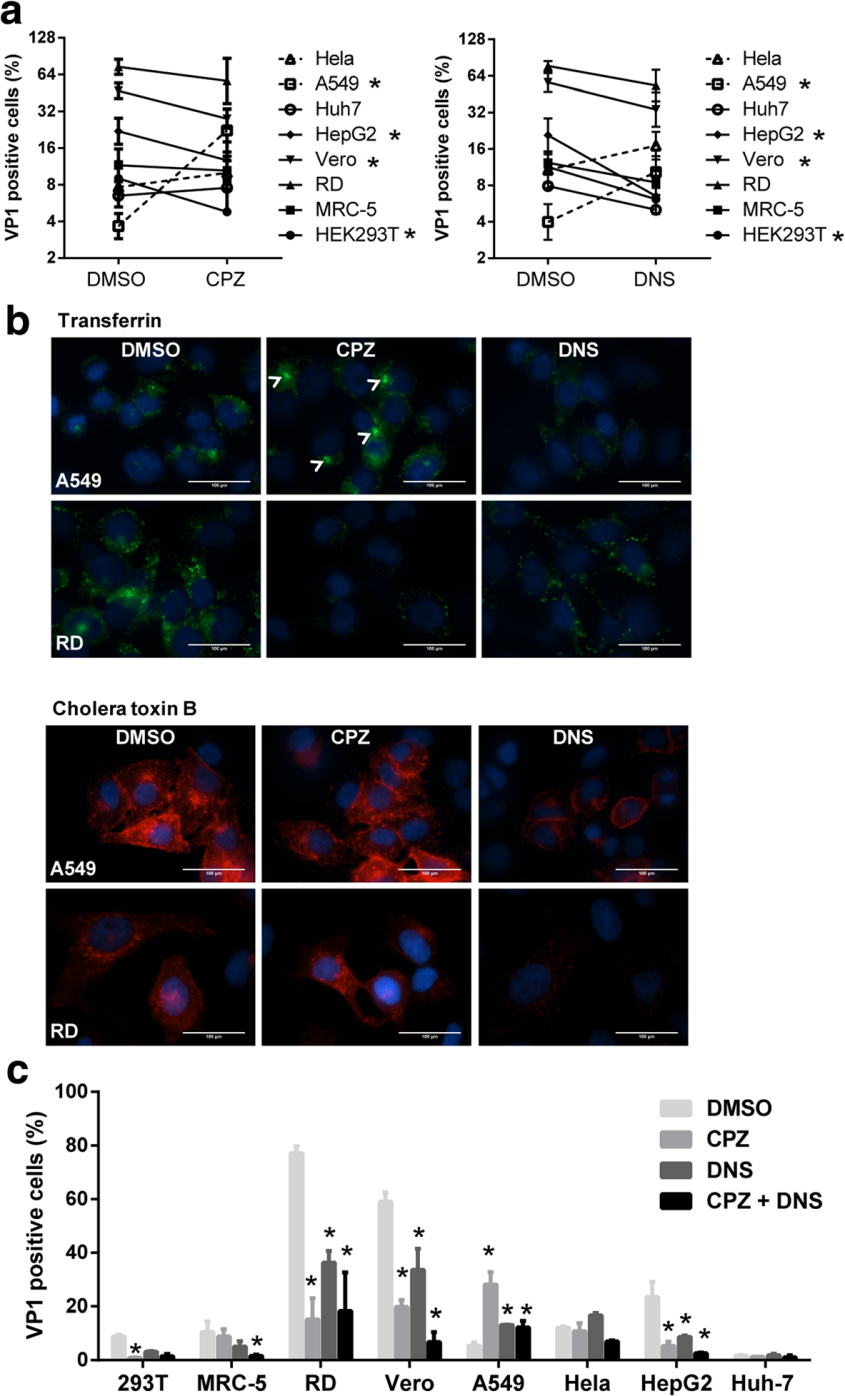
The effect of CPZ on EV71 infection is dynamin independent. **a**. Cells were infected by EV71 and treated with 20 μM CPZ or 80 μM DNS for 6 h (Vero, RD, MRC-5 and HEK293T, MOI = 1) or 12 h (Hela, A549, Huh7 and HepG2, MOI = 5). After removal of virus and the drugs, the cells were washed and continued to culture for 6 h or 12 h and then processed for flow cytometry. Means of three experiments with SD are shown. *, *p* < 0.05. **b**. A549 and RD cells were pretreated by 20 μM CPZ or 80 μM DNS for 2 h prior to incubation with AlexaFluor 488-conjugated transferrin (25 μg/ml) or AlexaFluor 594-conjugated cholera toxin B (CTxB, 10 μg/ml). After 15 min of incubation, cells were fixed and analyzed by immunofluorescent microscope. Arrow heads showed the bright puncta around nuclei. Scale bar, 100 μm. **c**. Various cells were infected by EV71 and treated by CPZ (20 μM), DNS (80 μM) or their combinations for 6 h (Vero, RD, MRC-5 and HEK293T, MOI = 1) or 12 h (Hela, A549, Huh7 and HepG2, MOI = 5). After removal of virus and the drug, cells were washed and continued to culture for 6 h or 12 h and then processed for flow cytometry. Means of three experiments with SD are shown. *, *p* < 0.05

Dynasore (DNS), an inhibitor of dynamin activity, could inhibit both clathrin- and caveolar-dependent endocytosis. However, shown in Fig. 2a, both CPZ and DNS could enhance EV71 infection in A549 cells, but not in other cells. Treatment by DNS also revealed a slightly increased infection in Hela cells. To assess the specificity and efficacy of endocytosis inhibitors, we performed the uptake assay of transferrin (through clathrin-mediated endocytosis) and cholera toxin B (CTxB, through caveolar-mediated endocytosis) under drug treatment. The results showed that CPZ specifically suppressed the uptake of transferrin but not CTxB in RD cells. Uptake of both transferrin and CTxB were inhibited by DNS in RD and A549 cells. However, an enhanced transferrin uptake was surprisingly observed in A549 cells after CPZ treatment, shown as many bright puncta around the cell nuclei. Meanwhile, no effect of CPZ on CTxB uptake was confirmed in A549 cells (Fig. 2b).

In order to investigate whether the enhanced EV71-infection by CPZ on A549 cells still depends on dynamin activity, we treated cells with combinations of CPZ and DNS. The results showed significantly enhanced infection in A549 cells after the treatment by CPZ, DNS or combinations. However, combined treatment caused most inhibition in other cells comparing to individual CPZ or DNS treatment (Fig. 2c).

### Characterization of CPZ effect on A549 cells revealed a slow EV71 infection kinetics.

**Fig. 3:**
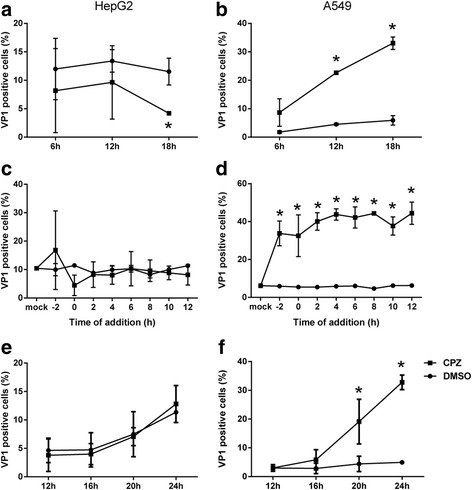
Characterization of the CPZ effect. **a-b**. HepG2 and A549 cells were infected by EV71 at an MOI of 5 in the presence of CPZ (20 μM) or DMSO for 6 h, 12 h and 18 h. After removal of virus and the drug, cells were washed and continued to culture until 24 h and then processed for flow cytometry. **c-d**. HepG2 and A549 cells were infected by EV71 at an MOI of 5. CPZ (20 μM) or DMSO was then added at −2, 0, 2, 4, 6, 8, 10, 12 hpi. At 24 hpi, cells were collected for VP-1 staining. The infectivity was measured as percentage of VP-1 positive cells. **e-f**. HepG2 and A549 cells were infected with EV71 at an MOI of 5 for 12 h, and then CPZ (20 μM) or DMSO was added. Cells were harvested at different time points (12 h, 16 h, 18 h and 24 h) afterward and subjected to VP-1 staining. Results were presented as the mean with SD from three independent experiments. *, *p* < 0.05. CPZ, filled square; DMSO, filled circle

To characterize the unique effect of CPZ on A549 cells, we investigated the effective time-frame of CPZ treatment on EV71 infection. Firstly, A549 or HepG2 cells were infected by EV71 in the presence or absence of CPZ for 6 h, 12 h and 18 h, and then EV71 infections were examined at 24 h. We noticed that the longer the incubation time was, the more EV71-infection in A549 cells resulted, no matter the presence or absence of CPZ treatment; while maximum infectivity in HepG2 could be achieved at 6 h and CPZ significantly inhibited EV71-infection in HepG2 cells at all time-points. The enhancement of infectivity by CPZ on A549 cells was also confirmed (Fig. 3a-b).

We next examined the effect of CPZ using time-of-addition experiments. First, we treated EV71-infected HepG2 and A549 cells by adding 20 μM CPZ at −2, 0, 2, 4, 6, 8, 10 and 12 h post-infection (hpi), where 0 hpi indicated time of virus inoculation. 24 h after infection, cells were collected and examined for infectivity. We found that 12 h treatment of CPZ enabled maximum EV71 infections in A549 cells, and prolonged incubation time did not result in more infectivity (Fig. 3c-d). Further probing the time beyond 12 hpi, we found reduced infectivity by shortening CPZ treatment, while CPZ treatment in HepG2 cells did not enhance EV71-infection at all (Fig. S2). Since maximum enhancement by CPZ was achieved during the last 12 h during a 24-h infection period, we next monitored the viral activity kinetics over this time frame. Our data showed no effect by CPZ treatment in HepG2 cells, but CPZ treatment led to an obviously increased infection in A549 cells over this period (Fig. 3e-f). All together, this data indicated slower viral infection kinetics (at least 12 h) in A549 cells than HepG2 cells (less than 6 h), and CPZ did not shortcut this process despite of overall enhancement of infectivity.

### CPZ enhanced the viral entry, and not viral translation or replication.

**Fig. 4:**
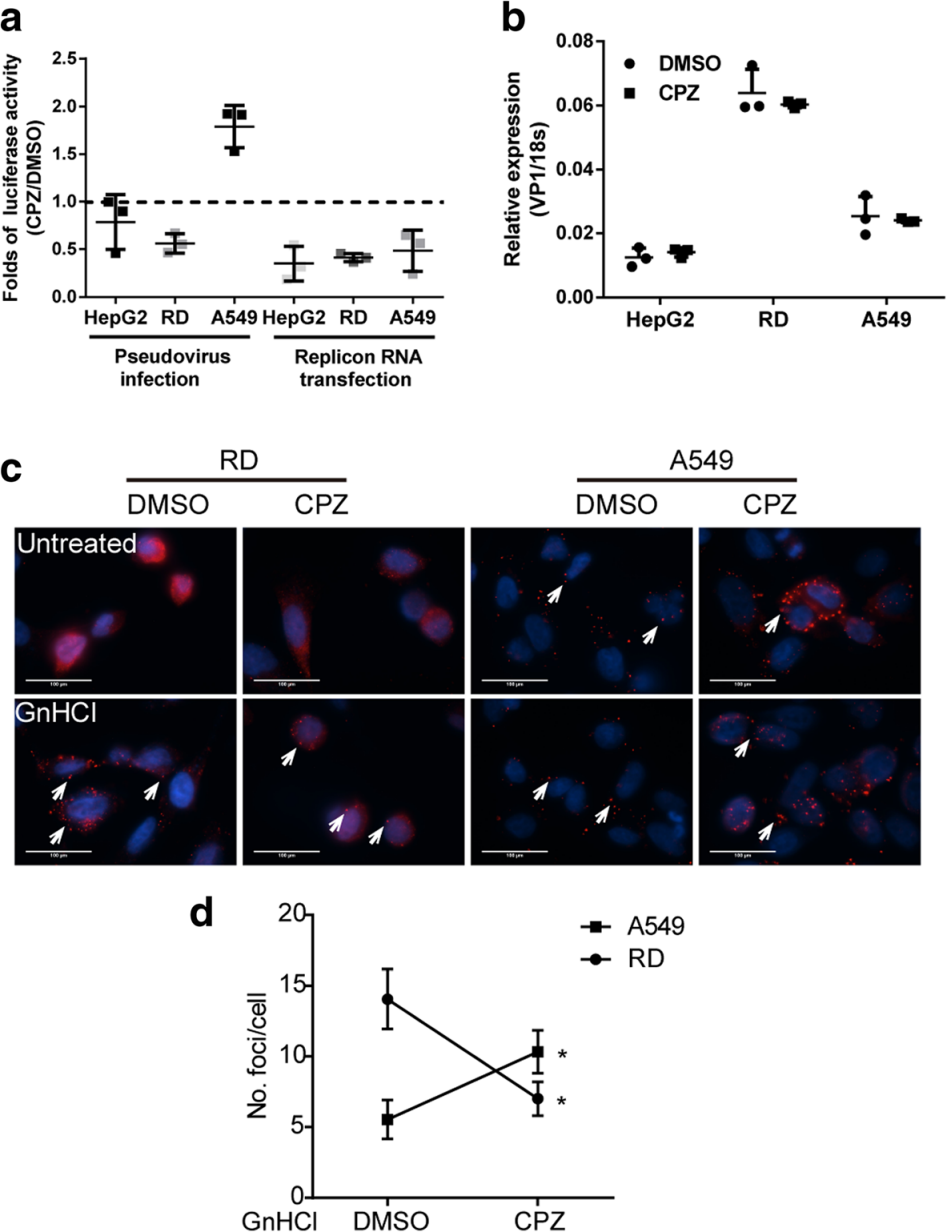
CPZ facilitates the entry step of EV71 infection. **a**. A549, RD and HepG2 cells were infected with EV71 pseudovirus or transfected with EV71 replicon RNA for 12 h before CPZ (20 μM) or DMSO treatment. Luciferase activity was quantified at 24 h post infection or transfection. The means with SD from three independent experiments each carried out in duplicate are shown. **b**. A549, RD and HepG2 cells were incubated with EV71 at an MOI of 50 and treated by CPZ or DMSO at 4 °C for 2 h. Then cells were washed extensively and lysed for viral RNA quantification with normalization to 18 s. Data shown are the means with SD from three independent experiments each carried out in duplicate. **c**. Immunofluorescence analysis was performed on RD and A549 cells infected with EV71 at an MOI of 10 (in RD) and 50 (in A549) in the presence or absence of 2.5 mM GnHCl. After 2 h incubation at 4 °C, cells were immediately shifted to 37 °C and treated by CPZ (20 μM) or DMSO. At 6 hpi, cells were washed extensively, and then fixed and stained with EV71 VP-1 antibody, and DAPI was used to visualize the nuclei. The images were one representative experiment out of three independent experiments. Arrow heads showed the VP-1 foci inside the cells. Scale bar, 100 μm. **d**. Frequency of VP-1 foci in each infected A549 and RD cell that were pretreated by GnHCl and then incubated with EV71 in the present of CPZ or DMSO. A paired Student’s *t* test was performed between the mean values in three independent experiments. *, *p* < 0.05

We speculated that CPZ enhanced EV71 entry into A549 cells. To test this possibility, we studied the effect of CPZ on EV71 replicon RNA replication and pseudotype virus infection on different cells. As shown in Fig. 4a, CPZ treatment increased almost 2-fold luciferase activity in A549 cells after EV71 pseudotype infection, while it did not affect or even decreased infectivity in RD and HepG2 cells. CPZ treatment also did not increase the replication or translation of EV71 replicon RNA when transfected into the three cell lines. Furthermore, no differences were observed in virus bindings after CPZ treatment (Fig. 4b). These results indicated that CPZ likely affected an early post-binding event in A549 cells but not a post-entry step, like the viral translation or replication.

To directly observe whether CPZ indeed enhanced EV71 entry into A549 cells, we monitored the entering viral capsids by VP-1 staining in cells with or without pretreatment of 2.5 mM guanidine hydrochloride (GnHCl), a known inhibitor of enterovirus RNA replication, before EV71 infection and CPZ treatment. We found that the VP-1 staining was decreased in RD cells and increased in A549 cells upon the treatment of CPZ. In the presence of GnHCl and absence of viral replication, many VP-1 puncta instead of diffusive staining was observed in RD cells, and CPZ significantly decreased the number of the puncta. In contrast, significantly more puncta was observed in A549 cells after CPZ treatment (Fig. 4c-d). In addition, we confirmed this data in A549 cells using UV-inactivated EV71 (Fig. S3). These results suggested that CPZ enhanced EV71 infection in A549 cells by directly modulating the entry step.

### The CPZ enhancement does not depend on dynamin and its associated endocytosis.

**Fig. 5:**
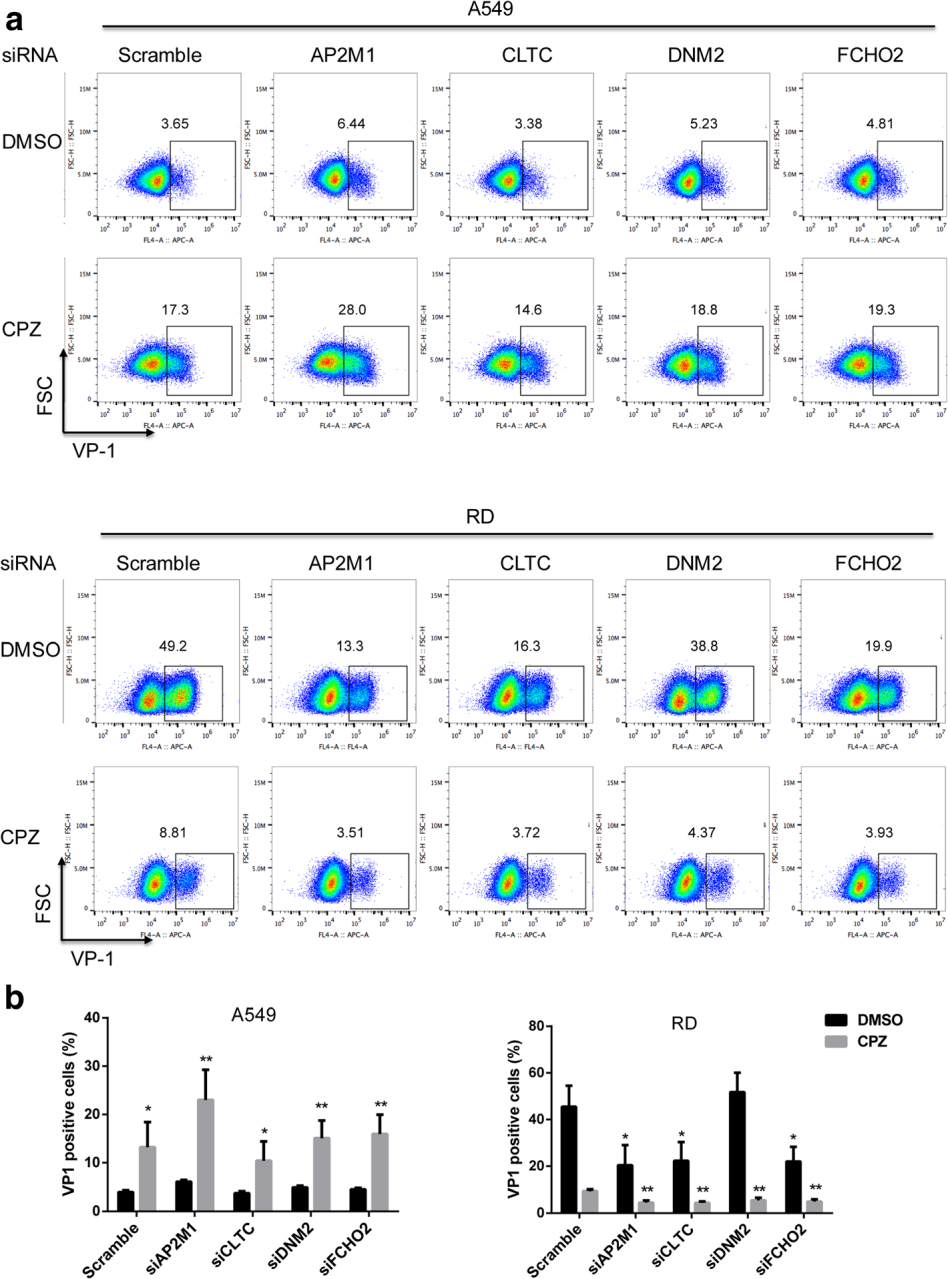
The effect of CPZ on EV71 infection after depletion of CME. **a**. A549 and RD cells were transfected with vary siRNA or scramble siRNA for 96 h. Then cells were infected with EV71 at an MOI of 1 in the presence of 20 μM CPZ or DMSO for 24 h (A549) or 6 h (RD). Flow cytometry was applied to determine the viral infectivity. The representative percentages of the infected cells were indicated. **b**. The bar plots showed the summary of three independent experiments. The significance was determined by paired Student’s *t* test by comparing CPZ treatment to DMSO control (A549) or gene-knockdown group to scramble group (RD). *p < 0.05; **p < 0.01

To examine the roles of clathrin-dependent endocytic pathway in the enhancement of EV71-infection by CPZ, we performed siRNA knockdown of the AP2M1, CLTC, DNM2 and FCHO2 (the reported crucial CME components [23]) in A549 and RD cells and tested the viral infectivity with additional CPZ treatment. Q-PCR assays confirmed more than 80% knockdown efficiency of AP2M1, CLTC, DNM2 and FCHO2 mRNAs (Fig. S4a-b). Significantly reduced protein levels were also shown by Western-blot (Fig. S4c). We observed significantly reduced EV71 infection after AP2M1, CLTC and FCHO2 knockdown in RD cells, and siRNA plus CPZ treatment exerted even more inhibition on EV71 infectivity. In contrast, EV71 infections in A549 cells were not significantly affected by siRNA knockdown of the targeted genes, and CPZ still enhanced EV71 infection in gene-knockdown A549 cells (Fig. 5a-b). Altogether, these data showed that EV71 infected A549 cells using a novel endocytic pathway which is independent of clathrin and dynamin, and this enhancing effect of CPZ did not require the functional components of clathrin or dynamin pathways.

### Coxackivirus A16 infected A549 cells using a dynamin-independent mechanism.

**Fig. 6:**
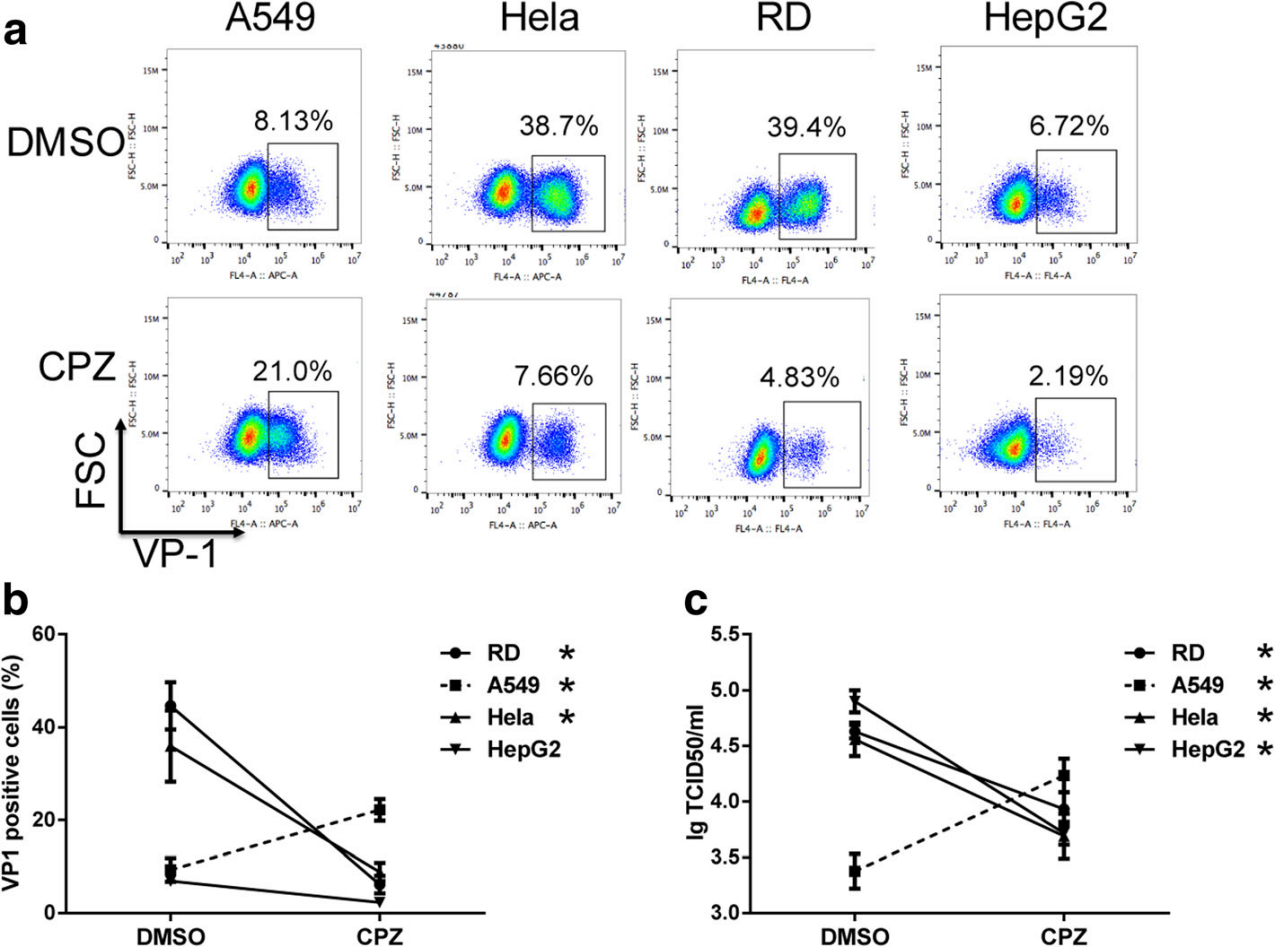
The effect of CPZ on CV-A16 infection. A549, Hela, RD and HepG2 cells were pretreated with CPZ (20 μM) or DMSO for 2 h. Then the cells were infected by CV-A16 at an MOI of 1. At 12 hpi (A549 and RD) or 24 hpi (HepG2 and Hela), flow cytometry was applied to determine the viral infectivity. The representative percentages of the infected cells were indicated (**a**). **b**. The plot showed the summary of three independent experiments. **c**. Virus titers in A549, RD, HepG2 and Hela cells were measured and shown in TCID50. The data were shown as means ± SD from three independent experiments. *, *p* < 0.05

We next determined whether the enhancement of CPZ is unique to EV71 infection. To address this, we sought to determine whether CPZ treatment could facilitate infection of another HFMD associated virus, CV-A16. Similar to that observed with EV71, treatment of CPZ enhanced infection of CV-A16 in A549 cells but rather inhibited viral infectivity in Hela, RD and HepG2 cells (Fig. 6a-b). In addition, CV-A16 viral growth was also increased in A549 cells but decreased in other cells after CPZ treatment (Fig. 6c). These data indicate that this novel endocytic pathway may be a conserved strategy among HFMD associated viruses to facilitate viral entry, and could be enhanced by CPZ.

## Discussion

EV71 is a prototype of *Enterovirus A* species which consist more than 20 serotypes, causing HFMD, herpangina, and other diseases in infants and young children [24]. Unlike *Enterovirus B* and *Enterovirus C* species which have been extensively investigated, the study of *Enterovirus A* is relatively few. Most of the knowledge about *Enterovirus A* came from the studies of EV71. Several molecules had been identified as potential EV71 receptors, however, only SCARB2 is widely distributed, capable of viral binding, viral internalization, and triggering uncoating [25]. Two dynamin-dependent endocytic pathways, the CME and CDE, were discovered to be utilized for EV71 entry into SCARB2 and PSGL-1 expressing cells respectively [4]. Here, expanding the study of EV71 in various cell lines, we surprisingly found that CME and dynamin inhibitors even enhanced EV71 infection in A549 cells, indicating an unknown dynamin-independent endocytic pathway by EV71. Clearly, this data increased our knowledge of *Enterovirus A*.

To our best knowledge, CPZ either inhibited or had no effect on viral entry in previous reports [15, 26], so the observation of enhancement of EV71 infection by CPZ is somewhat unusual and surprising. We confirmed that the enhanced EV71 entry was replication competent, since viral activities significantly increased, indicated by higher levels of eIF4G cleavage, viral nonstructural proteins, viral RNA and titers. So it is quite perplexed how CPZ affects EV71 infection in opposite ways in different cells. CPZ is an amphipathic molecule and binds to lipid bilayers; it had been reported to induce fusion of human red blood cells and viral envelopes, so we wandered that CPZ might modify the membrane properties [27, 28]. However, two facts disagreed with this hypothesis. First, CPZ was reported to affect membrane fluidity only at higher concentrations (mM) rather than μM range utilized here. Second, we did not observe that CPZ enhanced binding of virions to any target cells. CPZ also showed no direct effect on EV71 virions in pretreatment experiments. Another possibility is that CPZ may enhance other endocytic pathways due to a compensatory response after its inhibition of CME. Actually, the cross-regulation between different endocytic components were recently reported [29]. In this regard, we reasoned that CPZ resulted CME inhibition in A549 cells might positively affect other uncharacterized CME-independent endocytic pathways which were responsible for both the increased transferrin uptake and increased EV71 entry. Characterization of the exact steps of viral life cycle affected by CPZ also confirmed that the early entry step and not other processes were enhanced. Thus, our data supported that the endocytic system functioned uniquely in different cell types and another CME-independent pathway was responsible for EV71’s entry in A549 cells. Interestingly, CPZ was recently reported to be a strong autophagy inducer [30, 31]. As autophagy was found to be required for efficient EV71 replication [32–34], could CPZ positively affect EV71 activity through inducing autophagy? Although without clear evidence, we found that CPZ inhibited the activity of transfected EV71-subgenomic replicon RNA, suggesting CPZ did not enhance viral translation and replication. Therefore, our data dispute the potential involvement of autophagy which mainly affects post-entry activities. These findings also have implications for applying CPZ and its derivatives in antiviral therapies. CPZ is an FDA-approved antipsychotic drug used to treat schizophrenia and bipolar disorder. Due to its safety and its potency in inhibiting viral endocytosis, it had been attempted before for treating John Cunningham virus infections [14]. However, our data indicate that the effect of its application may be complicated since it may rather enhance viral infectivity at different tissues.

Our study reveals a novel clathrin and dynamin independent endocytic pathway for cellular entry of EV71 and CV-A16. Previous studies investigated limited number of cell lines, such as RD [11], SCARB2 expressing murine NIH-3 T3 and L929 cells [12], or Jurkat and PSGL-1-expressing L929 cells [13], and concluded that CME or CDE were indispensable for SCARB2- or PSGL-1 mediated EV71 entry. However, EV71/CV-A16 can infect various organs and cause a variety of diseases. They enter the host through fecal-oral route, spreading from mucosa, crossing epithelial barrier to the circulation, and then reaching to multiple tissues [35]. Therefore, a complete understanding of EV71 entry requires a detailed investigation of various cell types. Indeed, the closely related *Enterovirus B* viruses were found to use alternative surface receptors and internalize in receptor-limited cell types [36]. Another neurotropic virus, herpes simplex virus can use different endocytic route to infect Hela cells, cultured epiderm and neurons [37, 38]. Consistently, we found here that a novel entry pathway might be engaged by EV71 as well. Initially, we suspected this pathway was clathrin independent but still required dynamin. However, further investigation using dynamin inhibitors and siRNA certainly ruled out the involvement of dynamin. We are attempted to speculate that this novel endocytic pathway might mimic the one utilized by the *Enterovirus B* species which has been associated with micropinocytosis [36], due to their similarities with *Enterovirus A*. However, the exact mechanism of this new route, the receptor usages, the endocytic compartment for uncoating etc. are still not clear yet and certainly should be pursued next.

## Conclusion

In conclusion, we found novel evidence that endocytosis inhibitors may not inhibit but rather enhance EV71 infections in A549 cells. The mechanism is likely due to the effects on alternative endocytic routes, leading to increased viral entry. Present study clearly showed the existence of multiple endocytic mechanisms utilized by enteroviruses that cause HFMD.

## Additional file

Additional file 1: Figure S1. Pretreatment of EV71 by CPZ had no effect on subsequent infection in A549 cells. EV71 was pretreated by DMSO or CPZ (20 μM), and then infected A549 cells (MOI=5). The “0h” indicated the infection in the presence or absence of CPZ (20 μM) without CPZ pretreatment. 12 h later, virus was removed and VP-1 expression was examined at 24 h. Means of three experiments are shown. *, *p*<0.05. **Figure S2.** Characterization of the CPZ effect. HepG2 and A549 cells were infected with EV71 (MOI=5) for 12 h, and then CPZ (20 μM) or DMSO was added at indicated time points. 24 hpi, cells were subjected to VP-1 staining. CPZ, filled square; DMSO, filled circle. **Figure S3.** CPZ enhanced UV-inactivated EV71 uptake in A549 cells. a. A549 cells were incubated with EV71 or UV-inactive EV71 at an MOI of 50 at 4 °C for 2 h, and then shifted to 37 °C and treated by CPZ (20 μM) or DMSO. 6 hpi, cells were stained with VP-1 antibody (Red, VP-1; DAPI, nuclei). Scale bar, 100 μm. b. Frequency of VP-1 foci in infected A549 cell was compared using paired Student’s *t* test. *, *p*<0.05. **Figure S4.** The knockdown efficiency of CME in A549 and RD cells. a-b. The mRNA levels of targeted genes were measured by qPCR after 48 h transfection (normalized to 18s mRNA). c. The protein levels were detected by western blot at 96 h post-transfection. GAPDH was used as an internal control. The bar plots were summarized from three independent experiments. **Figure S5.** The effect of CPZ on EV71 infection in A549 cells. The full length blots of Fig. 1b. **Figure S6.** CPZ facilitates the entry step of EV71 infection. Part of the Fig. 4c image was enlarged to show the viral foci in A549 cells more clearly. (DOC 2327 kb)

## Abbreviations

CDE: Caveolar-dependent endocytic pathway
CME: Clathrin-mediated endocytosis
CPZ: Chlorpromazine
CTxB: Cholera toxin B
CV-A10: Coxackievirus A10
CV-A16: Coxackievirus A16
CV-A6: Coxackievirus A6
DNS: Dynasore
EV71: Human enterovirus 71
GnHCl: Guanidine hydrochloride
HFMD: Hand-Foot-and-Mouth Diseases
PSGL1: P-selectin glycoprotein ligand-1
SCARB2: Scavenger receptor class B, member 2
siRNAs: Small interfering RNAs
