# Supplementary material for: Enhanced human enterovirus 71 infection by endocytosis inhibitors reveals multiple entry pathways by enterovirus causing hand-foot-and-mouth diseases
Source: Virol J. 2018 Jan 3;15:1. doi: 10.1186/s12985-017-0913-3 (PMC5751926; doi:10.1186/s12985-017-0913-3)

**Supplementary Figures and legends**

**
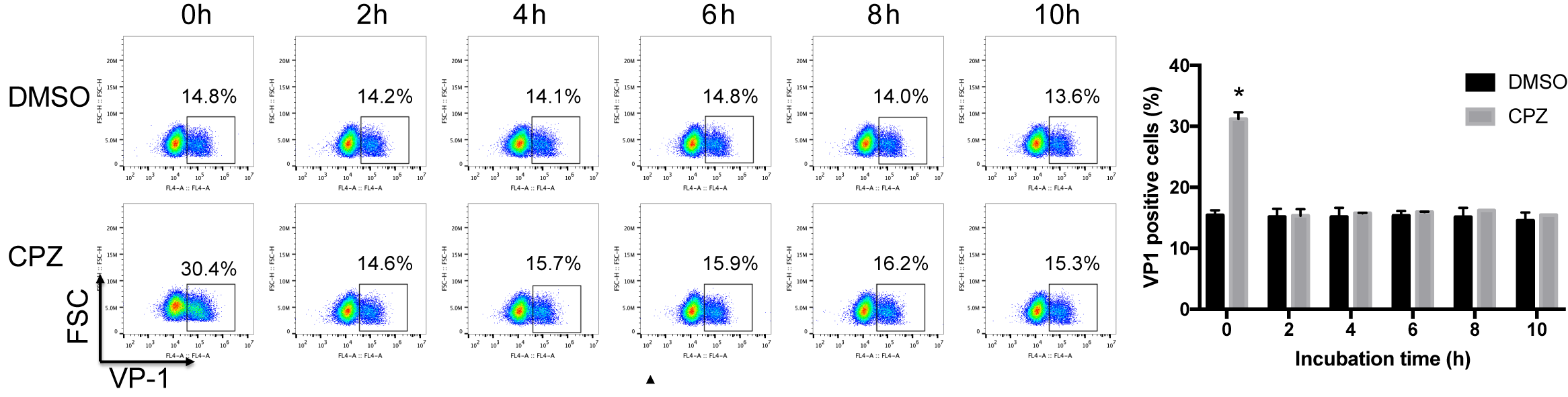
**

**Figure S1. Pretreatment of EV71 by CPZ had no effect on subsequent infection in A549 cells.** EV71 was pretreated by DMSO or CPZ (20μM) for different time (2, 4, 6, 8, 10h), and then added to A549 cells (MOI=5). The “0h” indicated no CPZ pretreatment, and then the viral infection was conducted in the presence or absence of CPZ (20μM). 12h later, virus was removed, and the cells were washed and continued to culture for 12h. Then the cells were processed for flow cytometry. Means of three experiments with SD are shown. *, *p*<0.05.

**
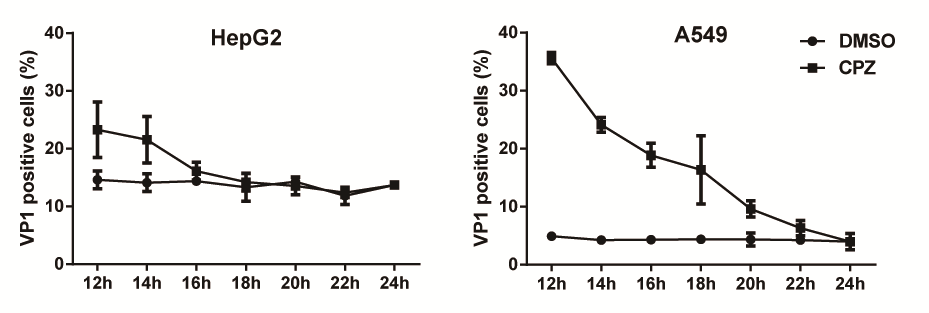
**

**Figure S2. Characterization of the CPZ effect.** HepG2 and A549 cells were infected with EV71 at an MOI of 5 for 12h, and then CPZ (20μM) or DMSO was added at indicated time points (12, 14, 16, 18, 20, 22 hpi). At 24 hpi, cells were harvested and subjected to VP-1 staining. CPZ, filled square; DMSO, filled circle.


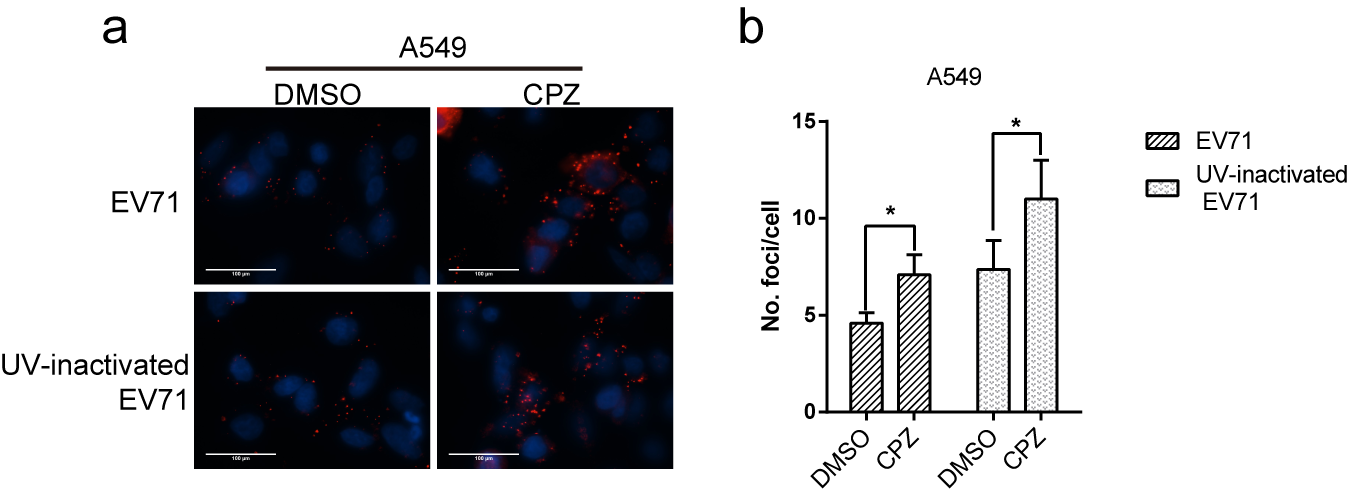


**Figure S3. CPZ enhanced UV-inactivated EV71 uptake in A549 cells.** a. A549 cells were incubated with EV71 or UV-inactive EV71 at an MOI of 50 at 4°C for 2h. Then the cells were immediately shifted to 37°C and treated by CPZ (20μM) or DMSO. At 6 hpi, cells were fixed and stained with mouse anti-EV71 VP-1 antibody and AlexFluor 594 goat anti-mouse IgG. DAPI was used to visualize the nuclei. Scale bar, 100μm. b. Frequency of VP-1 foci in each infected A549 cell after incubated with EV71 or UV-inactivated EV71 during CPZ treatment or DMSO control. A paired Student’s *t* test was performed between the mean values in three independent experiments. *, *p*<0.05.


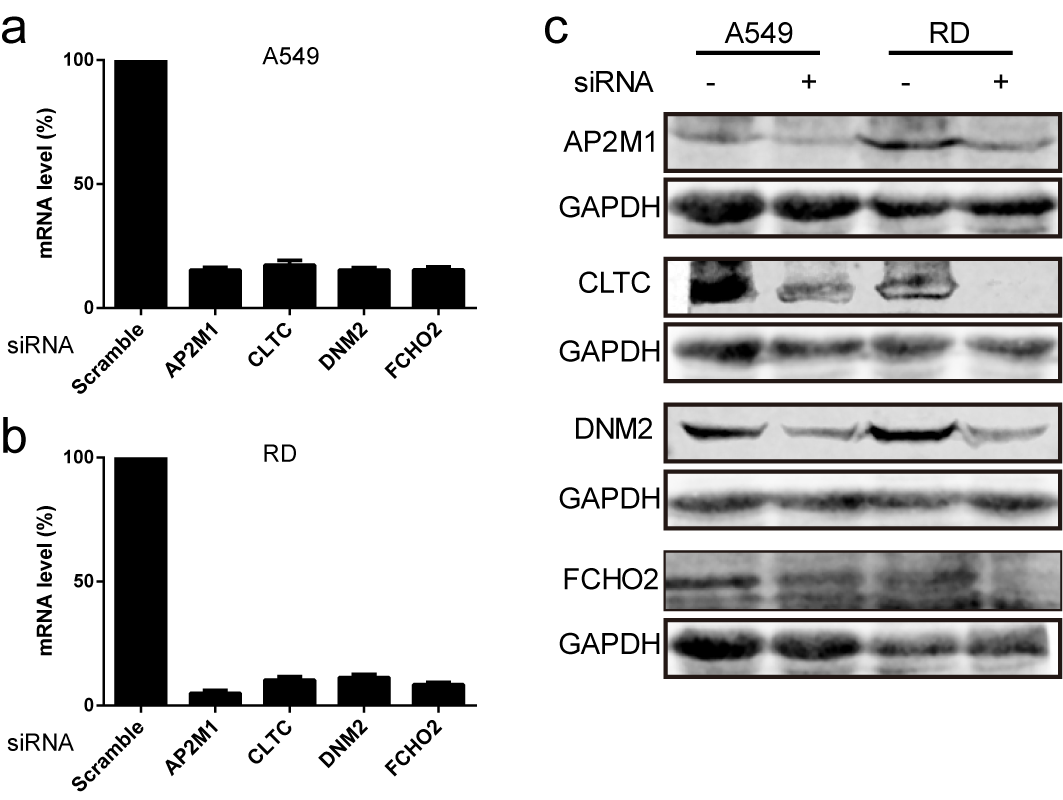


**Figure S4. The knockdown efficiency of CME in A549 and RD cells.** a-b. A549 and RD cells were transfected with vary siRNA or scramble siRNA. The mRNA levels of targeted genes were measured by qPCR after 48h transfection with normalization to 18s RNA. c. The protein levels were detected by western blot at 96h post-transfection. GAPDH was used as an internal control. The bar plots were shown as the means with SD from three independent experiments. The blot was shown as one representative out of three independent experiments.


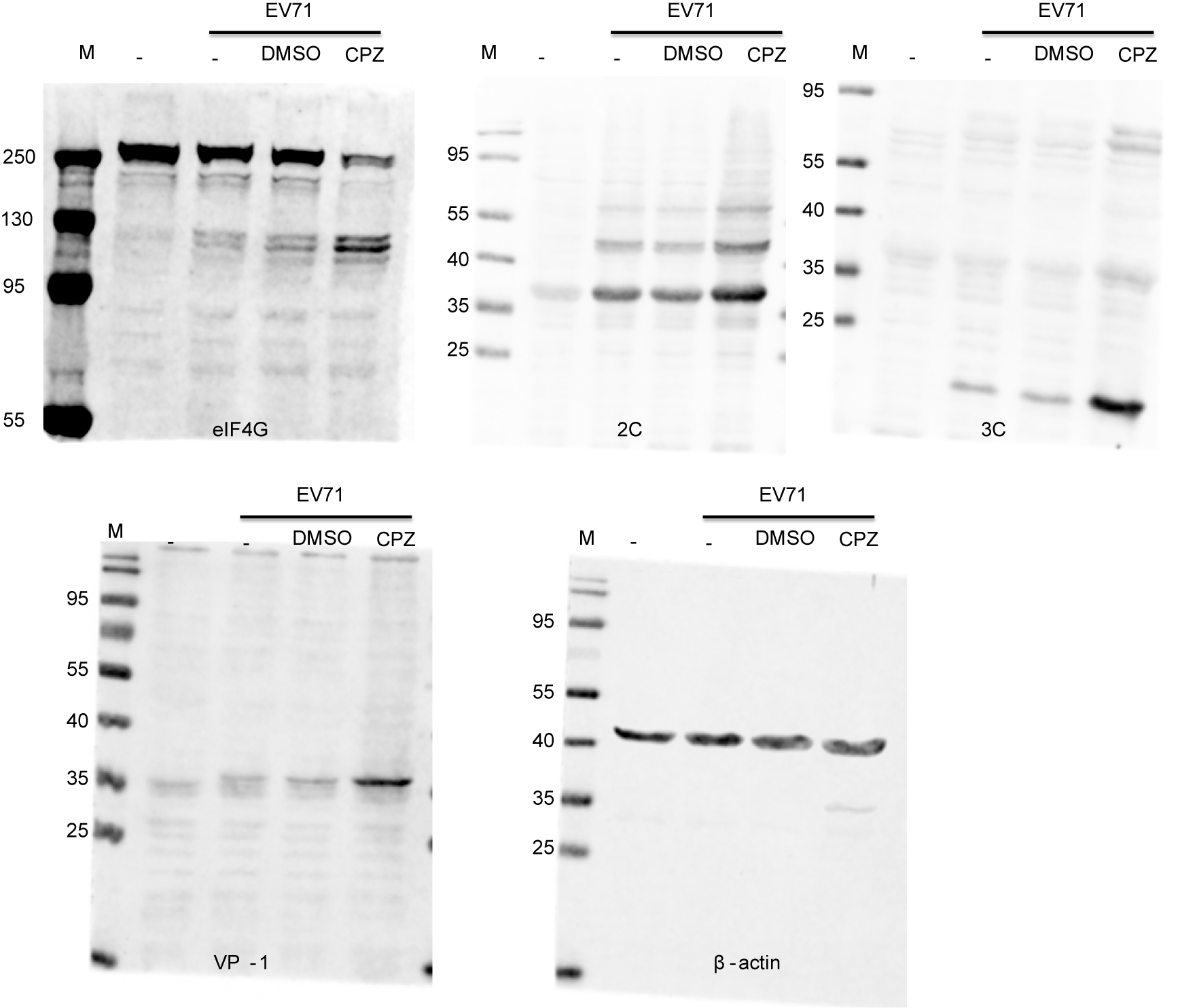


**Figure S5. The effect of CPZ in EV71 infection in A549 cells.** A549 cells were infected with EV71 at an MOI of 5 for 12h and then CPZ or DMSO was added. At 24 hpi, cells were lysed, and eIF4G cleavage, EV71- 2C, 3C and VP-1 were determined by Western blot.


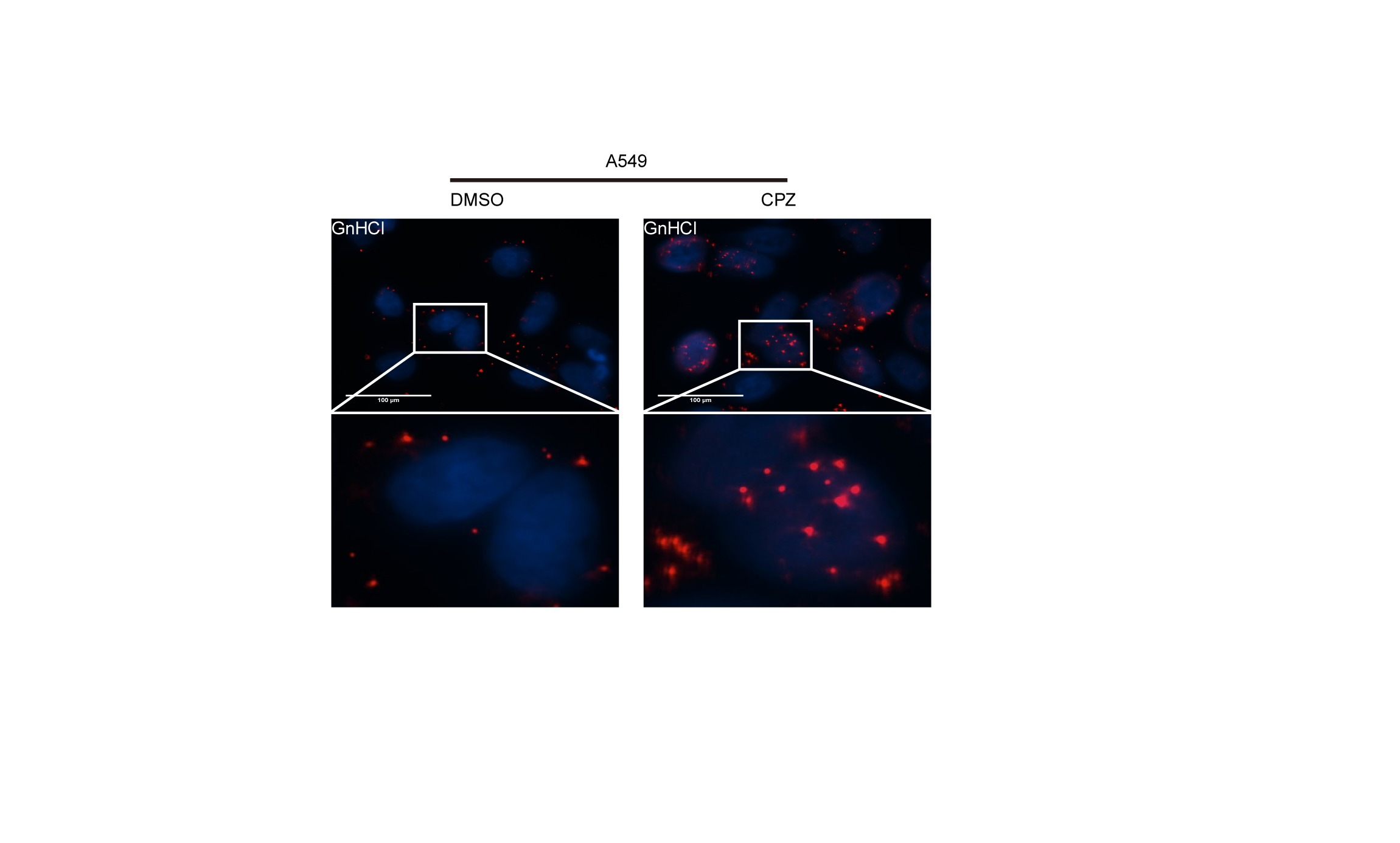


**Figure S6.** **CPZ facilitates the entry step of EV71 infection.** Part of the Figure 4c image was enlarged to show the viral foci in A549 cells more clearly.

**Supplementary Table**


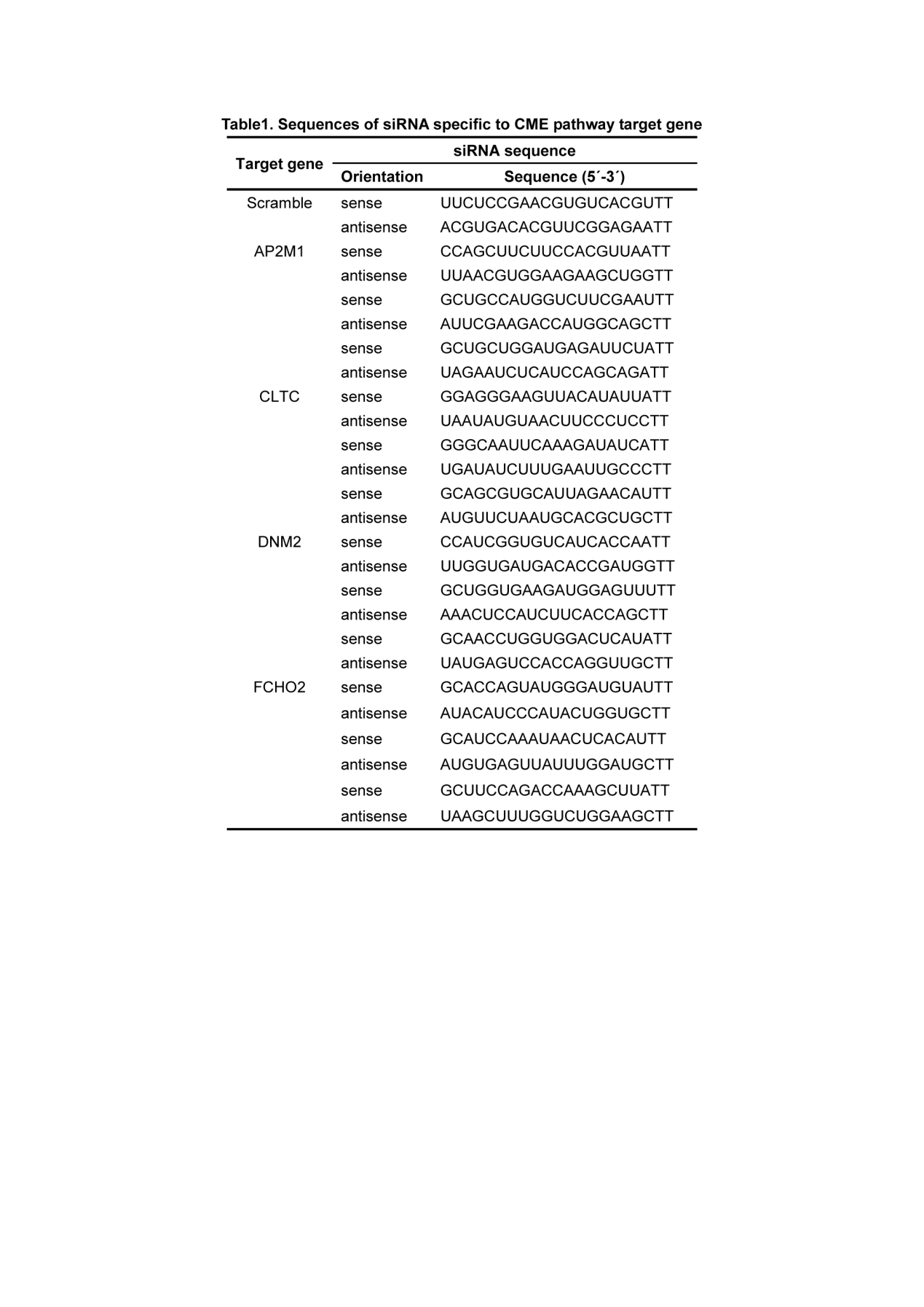

Supplement: Additional file 1: Figure S1. — Pretreatment of EV71 by CPZ had no effect on subsequent infection in A549 cells. EV71 was pretreated by DMSO or CPZ (20 μM), and then infected A549 cells (MOI=5). The “0h” indicated the infection in the presence or absence of CPZ (20 μM) without CPZ pretreatment. 12 h later, virus was removed and VP-1 expression was examined at 24 h. Means of three experiments are shown. *, p<0.05. Figure S2. Characterization of the CPZ effect. HepG2 and A549 cells were infected with EV71 (MOI=5) for 12 h, and then CPZ (20 μM) or DMSO was added at indicated time points. 24 hpi, cells were subjected to VP-1 staining. CPZ, filled square; DMSO, filled circle. Figure S3. CPZ enhanced UV-inactivated EV71 uptake in A549 cells. a. A549 cells were incubated with EV71 or UV-inactive EV71 at an MOI of 50 at 4 °C for 2 h, and then shifted to 37 °C and treated by CPZ (20 μM) or DMSO. 6 hpi, cells were stained with VP-1 antibody (Red, VP-1; DAPI, nuclei). Scale bar, 100 μm. b. Frequency of VP-1 foci in infected A549 cell was compared using paired Student’s t test. *, p<0.05. Figure S4. The knockdown efficiency of CME in A549 and RD cells. a-b. The mRNA levels of targeted genes were measured by qPCR after 48 h transfection (normalized to 18s mRNA). c. The protein levels were detected by western blot at 96 h post-transfection. GAPDH was used as an internal control. The bar plots were summarized from three independent experiments. Figure S5. The effect of CPZ on EV71 infection in A549 cells. The full length blots of Fig. 1b. Figure S6. CPZ facilitates the entry step of EV71 infection. Part of the Fig. 4c image was enlarged to show the viral foci in A549 cells more clearly. (DOC 2327 kb) [file 12985_2017_913_MOESM1_ESM.doc]
